# Supplementary material for: A systematic review comparing the performance of alternative blackfly (Simulium) trapping methods against the standard human landing catch (HLC) for onchocerciasis surveillance
Source: PLoS Negl Trop Dis. 2026 May 18;20(5):e0014359. doi: 10.1371/journal.pntd.0014359 (PMC13197077; doi:10.1371/journal.pntd.0014359)
Supplement: S1 Table — The PRISMA Checklist is licensed under a CCBY 4.0 license (https://creativecommons.org/licenses/by/4.0/deed.en) and attributed to Page MJ, McKenzie JE, Bossuyt PM, Boutron I, Hoffmann TC, Mulrow CD, et al. The PRISMA 2020 statement: an updated guideline for reporting systematic reviews. BMJ 2021;372:n71, https://doi.org/10.1136/bmj.n71. (DOCX) [file pntd.0014359.s001.docx]

| **Section and Topic** | **Item #** | **Checklist item** | **Location where item is reported** |
| --- | --- | --- | --- |
| **TITLE** | | |  |
| Title | 1 | Identify the report as a systematic review. | Title page: “A systematic review comparing the performance of alternative blackfly (*Simulium*) trapping methods against the standard human landing catch (HLC) for onchocerciasis surveillance” (manuscript title). **[Page 1]** |
| **ABSTRACT** | | |  |
| Abstract | 2 | See the PRISMA 2020 for Abstracts checklist. | Structured abstract (Abstract section) **→** Includes background, objective, information sources (Scopus, PubMed, Web of Science, expert list), eligibility criteria, number of records screened and included (166 identified; 13 included; 79 comparisons), registration (PROSPERO CRD420261294895), narrative synthesis approach (no meta-analysis due to heterogeneity), key findings (75.9% less effective), limitations (heterogeneity and incomplete statistical testing), and conclusions. **[Page 2]** |
| **INTRODUCTION** | | |  |
| Rationale | 3 | Describe the rationale for the review in the context of existing knowledge. | Background section (paragraphs 1–3) describing HLC limitations, need for alternatives and rationale for this synthesis. **[Page 5]** |
| Objectives | 4 | Provide an explicit statement of the objective(s) or question(s) the review addresses. | End of Introduction: “**This systematic review aims to evaluate the performance of alternative blackfly trapping methods in comparison to the standard HLC across different epidemiological contexts. [Page 6]** |
| **METHODS** | | |  |
| Eligibility criteria | 5 | Specify the inclusion and exclusion criteria for the review and how studies were grouped for the syntheses. | Methods **→** **Table 2: Inclusion and exclusion criteria**; PROSPERO entry (Study design, Included/Excluded). Grouping described in Methods (comparisons counted by trap type; synthesis narrative). **[Page 7]** |
| Information sources | 6 | Specify all databases, registers, websites, organisations, reference lists and other sources searched or consulted to identify studies. Specify the date when each source was last searched or consulted. | Methods **→** **Literature search**: Scopus, PubMed, Web of Science (searched 03 July 2024; updated 17 December 2025). Expert-provided list (03 July 2024). (Reported in Methods and in PROSPERO registration citation). **[Page 6]** |
| Search strategy | 7 | Present the full search strategies for all databases, registers and websites, including any filters and limits used. | Methods **→** **Table 1** gives keywords/search terms and database search strings **[Page 6]** |
| Selection process | 8 | Specify the methods used to decide whether a study met the inclusion criteria of the review, including how many reviewers screened each record and each report retrieved, whether they worked independently, and if applicable, details of automation tools used in the process. | Methods **→** **Screening and review**: Title/abstract screening done independently by two investigators (IK & AM) using Rayyan; discrepancies resolved with FH; full-text review conducted (IK). (Reported in Methods; consistent with PROSPERO). **[Page 7]** |
| Data collection process | 9 | Specify the methods used to collect data from reports, including how many reviewers collected data from each report, whether they worked independently, any processes for obtaining or confirming data from study investigators, and if applicable, details of automation tools used in the process. | Methods **→ Data extraction**: Data extraction performed by one reviewer (IK) into Excel (Reported in Methods; consistent with PROSPERO). **[Page 8]** |
| Data items | 10a | List and define all outcomes for which data were sought. Specify whether all results that were compatible with each outcome domain in each study were sought (e.g. for all measures, time points, analyses), and if not, the methods used to decide which results to collect. | Methods **→** **Data extraction** and Tables 6 & 7: outcomes extracted include total catch, mean hourly catch, mean daily catch, monthly biting rate **[Page 8]** |
|  | 10b | List and define all other variables for which data were sought (e.g. participant and intervention characteristics, funding sources). Describe any assumptions made about any missing or unclear information. | Methods **→ Data extraction**: study design; location; year; season; trap rotation; number of traps/collectors; proximity; species; trap type; statistical methods. PROSPERO also lists data items. For missing data, authors were not contacted **[Page 8]** |
| Study risk of bias assessment | 11 | Specify the methods used to assess risk of bias in the included studies, including details of the tool(s) used, how many reviewers assessed each study and whether they worked independently, and if applicable, details of automation tools used in the process. | **Prespecified in PROSPERO**: no formal risk of bias assessment will be conducted.  Methods **→ Risk of bias assessment** states that: no formal risk of bias assessment was conducted **[Page 9]** |
| Effect measures | 12 | Specify for each outcome the effect measure(s) (e.g. risk ratio, mean difference) used in the synthesis or presentation of results. | Methods → **Data extraction**: extracted the measure as reported by original studies (total catch, mean hourly/daily catch, monthly biting rate). No pooled effect measure was calculated **[Page 8]** |
| Synthesis methods | 13a | Describe the processes used to decide which studies were eligible for each synthesis (e.g. tabulating the study intervention characteristics and comparing against the planned groups for each synthesis (item #5)). | Methods → **Synthesis**: narrative synthesis was conducted due to heterogeneity; studies grouped descriptively by trap type, habitat and season. Tables 3–7 tabulate study/trap characteristics to support grouping. **[Page 9]** |
|  | 13b | Describe any methods required to prepare the data for presentation or synthesis, such as handling of missing summary statistics, or data conversions. | Methods → **Synthesis**: extracted metrics were used as reported, no conversions were performed; missing data recorded as missing; no imputation. **[Page 9]** |
|  | 13c | Describe any methods used to tabulate or visually display results of individual studies and syntheses. | **Results/Tables**: Table 3 (study characteristics), Table 4 (habitat/season), Table 5 (EWT versions), Table 6 (outcomes), Table 7 (findings); Figure 2 PRISMA flowchart; Figures described in Results. **[Pages 8, 10-14]** |
|  | 13d | Describe any methods used to synthesize results and provide a rationale for the choice(s). If meta-analysis was performed, describe the model(s), method(s) to identify the presence and extent of statistical heterogeneity, and software package(s) used. | Methods **→** **Synthesis**: Narrative synthesis chosen due to heterogeneity; **No meta-analysis performed. [Page 9]** |
|  | 13e | Describe any methods used to explore possible causes of heterogeneity among study results (e.g. subgroup analysis, meta-regression). | Methods **→** **Synthesis**: No formal subgroup/meta-regression planned (PROSPERO stated no subgroup analyses). Narrative stratification by trap type, habitat and season where data permit (stated in Methods and used in Results). **[Page 9]** |
|  | 13f | Describe any sensitivity analyses conducted to assess robustness of the synthesized results. | Methods / PROSPERO: No sensitivity analyses planned (stated in PROSPERO and Methods; narrative synthesis only). **[Page 9]** |
| Reporting bias assessment | 14 | Describe any methods used to assess risk of bias due to missing results in a synthesis (arising from reporting biases). | Methods **→** **Synthesis**: Formal assessment not feasible due to small number and heterogeneity **[Page 9]** |
| Certainty assessment | 15 | Describe any methods used to assess certainty (or confidence) in the body of evidence for an outcome. | Methods / PROSPERO **→** **Certainty of evidence**: **No formal GRADE** assessment planned or performed due to heterogeneity. **[Page 9]** |
| **RESULTS** | | |  |
| Study selection | 16a | Describe the results of the search and selection process, from the number of records identified in the search to the number of studies included in the review, ideally using a flow diagram. | Results **→** **Figure 2** Flow diagram and Results text: “From 166 records, 62 were screened, 13 studies included” (printed in Results/Abstract). **[Page 8]** |
|  | 16b | Cite studies that might appear to meet the inclusion criteria, but which were excluded, and explain why they were excluded. | Results **→** Exclusions at full-text stage are shown in **Figure 2** **[Page 8]** |
| Study characteristics | 17 | Cite each included study and present its characteristics. | Results **→** The characteristics of each included study are summarised in **Tables 3–6**. Each of the 13 included studies is cited in the text and in Table 6. **[Page 10-13]** |
| Risk of bias in studies | 18 | Present assessments of risk of bias for each included study. | Methods**→ Risk of bias approach** and PROSPERO. Risk of bias was not formally assessed. Methodological limitations of included studies are described narratively in the Results and Discussion sections. **[Page 9]** |
| Results of individual studies | 19 | For all outcomes, present, for each study: (a) summary statistics for each group (where appropriate) and (b) an effect estimate and its precision (e.g. confidence/credible interval), ideally using structured tables or plots. | Results → **Tables 6 & 7** present outcomes and findings from each comparison (total catch, mean daily/hourly, monthly biting rate). Where original studies reported statistical estimates, these are recorded in extracted tables. **[Page 12-14]** |
| Results of syntheses | 20a | For each synthesis, briefly summarise the characteristics and risk of bias among contributing studies. | Results **→** Narrative synthesis paragraphs summarise trap types, habitats, seasons, and methodological heterogeneity. **[Page 11]** |
|  | 20b | Present results of all statistical syntheses conducted. If meta-analysis was done, present for each the summary estimate and its precision (e.g. confidence/credible interval) and measures of statistical heterogeneity. If comparing groups, describe the direction of the effect. | Methods and Abstract **→ No statistical syntheses/meta-analysis performed** (). Results present descriptive proportions (e.g., 75.9% of comparisons where alternative traps were less effective) and narrative findings (Tables 6–7). **[Pages 2, 9]** |
|  | 20c | Present results of all investigations of possible causes of heterogeneity among study results. | No formal heterogeneity analyses were performed. Narrative exploration by trap type, habitat and season is presented in Results (see sections describing EWT findings, Loum et al. examples). **[Page 11]** |
|  | 20d | Present results of all sensitivity analyses conducted to assess the robustness of the synthesized results. | No sensitivity analyses were performed (stated in Methods/PROSPERO). |
| Reporting biases | 21 | Present assessments of risk of bias due to missing results (arising from reporting biases) for each synthesis assessed. | **Not formally assessed** due to small study number and heterogeneity |
| Certainty of evidence | 22 | Present assessments of certainty (or confidence) in the body of evidence for each outcome assessed. | **No formal GRADE**; Results/Discussion include narrative judgement that overall certainty is low–moderate due to heterogeneity, incomplete reporting and frequent absence of statistical testing **[Page 15-16]** |
| **DISCUSSION** | | |  |
| Discussion | 23a | Provide a general interpretation of the results in the context of other evidence. | Discussion **→** interpretation, comparison with other evidence, explanation of the dominance of HLC and limitations of alternatives. **[Page 15-16]** |
|  | 23b | Discuss any limitations of the evidence included in the review. | Discussion **→** heterogeneity, inconsistent reporting, species-level issues and lack of statistical testing discussed. **[Page 15-16]** |
|  | 23c | Discuss any limitations of the review processes used. | Methods & Discussion **→** PROSPERO and Methods indicate single-extractor approach and prespecified no formal risk of bias assessment. Last paragraph of the discussion expounds further on limitations. **[Page 16]** |
|  | 23d | Discuss implications of the results for practice, policy, and future research. | Discussion & Conclusion **→** implications for surveillance policy, research priorities on trap standardisation and design, and ethical considerations of HLC. **[Page 16]** |
| **OTHER INFORMATION** | | |  |
| Registration and protocol | 24a | Provide registration information for the review, including register name and registration number, or state that the review was not registered. | Methods **→** PROSPERO registration CRD420261294895. See Methods and PROSPERO citation (reference 23) **[Page 6]** |
|  | 24b | Indicate where the review protocol can be accessed, or state that a protocol was not prepared. | Methods **→** PROSPERO record URL provided in manuscript (citation, reference 23). **[Page 6 & Page 20]** |
|  | 24c | Describe and explain any amendments to information provided at registration or in the protocol. | PROSPERO record shows initial registration; No amendments reported. |
| Support | 25 | Describe sources of financial or non-financial support for the review, and the role of the funders or sponsors in the review. | Funding/Grant information section **→** Gates Foundation grant INV-037397; Health Data Research UK support noted. Funders had no role in design/conduct/write-up |
| Competing interests | 26 | Declare any competing interests of review authors. | Competing interests section **→ “The authors declare no competing interests.” [Page 18]** |
| Availability of data, code and other materials | 27 | Report which of the following are publicly available and where they can be found: template data collection forms; data extracted from included studies; data used for all analyses; analytic code; any other materials used in the review. | Data & software availability section **→** Zenodo DOI: <https://doi.org/10.5281/zenodo.18631036> (extracted dataset). **[Page 18]** |

*From:*  Page MJ, McKenzie JE, Bossuyt PM, Boutron I, Hoffmann TC, Mulrow CD, et al. The PRISMA 2020 statement: an updated guideline for reporting systematic reviews. BMJ 2021;372:n71. doi: 10.1136/bmj.n71. This work is licensed under CC BY 4.0. To view a copy of this license, visit <https://creativecommons.org/licenses/by/4.0/>
