# Supplementary material for: A systematic review comparing the performance of alternative blackfly (Simulium) trapping methods against the standard human landing catch (HLC) for onchocerciasis surveillance
Source: PLoS Negl Trop Dis. 2026 May 18;20(5):e0014359. doi: 10.1371/journal.pntd.0014359 (PMC13197077; doi:10.1371/journal.pntd.0014359)
Supplement: Attachment — Submitted filename: Response to Reviewers.docx [file pntd.0014359.s003.docx]

**Response to reviewers for feedback on “A systematic review comparing the performance of alternative blackfly (*Simulium*) trapping methods against the standard human landing catch (HLC) for onchocerciasis surveillance”**

We thank the three reviewers and the editors for their positive feedback and constructive suggestions for improving our manuscript. We respond to these, as requested, point-by-point in the bullet points below.

**Journal Requirements**

- We have addressed points 1, 2, 3 and 5 in the editorial manager.

4) We notice that your supplementary Tables are included in the manuscript file. Please remove them and upload them with the file type 'Supporting Information'. Please ensure that each Supporting Information file has a legend listed in the manuscript after the references list.

- Please note that our Tables (1-7) are intended to be presented in the main text, in the approximate position where we have inserted them. We do not intend for them to be Supporting Information. Therefore, we have left all the Tables in their current positions, following the instructions in the PLoS NTD guidelines for tables: “*Place each table in your manuscript file directly after the paragraph in which it is first cited (read order). Do not submit tables in a separate file(s).*” (<https://journals.plos.org/plosntds/s/tables>). We have one Table that is Supplementary Information (S1 PRISMA Checklist), which we have uploaded separately and provided a legend for at the end of the manuscript. Please advise if this requires further edits.

6) Figure 1: Please confirm (a) that you are the photographer; or (b) provide written permission from the photographer to publish the photo(s) under our CC BY 4.0 license.

- We are not the photographers. In the [‘Acknowledgements’ section, we provide the appropriate](https://eur03.safelinks.protection.outlook.com/?url=https%3A%2F%2Fevents.rspb.org.uk%2Fevents%2F147377&data=05%7C02%7Cf.m.hawkes%40greenwich.ac.uk%7Cbc57f7704c944b9dfe8708de96d6943b%7C3516f40a5ae94956bbab395162e589ce%7C0%7C0%7C639114045548348572%7CUnknown%7CTWFpbGZsb3d8eyJFbXB0eU1hcGkiOnRydWUsIlYiOiIwLjAuMDAwMCIsIlAiOiJXaW4zMiIsIkFOIjoiTWFpbCIsIldUIjoyfQ%3D%3D%7C0%7C%7C%7C&sdata=Pp28UsmEuBBlKi0DKO5mMVFo2eyWo2V4%2BcahFElo4yM%3D&reserved=0) licences [for re-use of these images, as well as details of how the images have been handled.](https://eur03.safelinks.protection.outlook.com/?url=https%3A%2F%2Fevents.rspb.org.uk%2Fevents%2F147377&data=05%7C02%7Cf.m.hawkes%40greenwich.ac.uk%7Cbc57f7704c944b9dfe8708de96d6943b%7C3516f40a5ae94956bbab395162e589ce%7C0%7C0%7C639114045548364992%7CUnknown%7CTWFpbGZsb3d8eyJFbXB0eU1hcGkiOnRydWUsIlYiOiIwLjAuMDAwMCIsIlAiOiJXaW4zMiIsIkFOIjoiTWFpbCIsIldUIjoyfQ%3D%3D%7C0%7C%7C%7C&sdata=GUoMdH5FIXtrExMvkoqF3l%2BYQlZAK710Cbw1ULvUFfA%3D&reserved=0) All the images we reproduce are available in the public domain under CC BY 4.0 licence (<https://creativecommons.org/licenses/by/4.0/>), meaning we can “Share — copy and redistribute the material in any medium or format for any purpose, even commercially. Adapt — remix, transform, and build upon the material for any purpose, even commercially”, provided we follow the terms of Attribution “You must give appropriate credit, provide a link to the license, and indicate if changes were made. You may do so in any reasonable manner, but not in any way that suggests the licensor endorses you or your use.” To abide by these terms, in the ‘Acknowledgements’ section, we included the credit (reference cited), a link to the licence (as above) and described how the image was handled. If the journal would like us to present compliance with the CC BY 4.0 license to reproduce these images in an alternative format, please advise and we will adjust accordingly.

**Reviewers’ Comments:**

**Methods:**

Reviewer #1:

Line 115. Can probably just omit the clause before the comma and start with We conducted

- Actioned

Line 127. Because half of the expert-suggested references did not appear in the literature search, this begs the question – how many other relevant references were missed with the search terms/databases used? How did the authors verify that their search was comprehensive?

- Thank you for highlighting; our description here was unclear and we have rephrased to clarify. The list of expert-suggested material included 6 items that were not identified by the original search (which produced 160 records). The 6 expert-suggested items were then added to those from the formal database searches at the “identification step” (shown in Figure 2). This resulted in 166 records that were screened for inclusion. Ultimately, these 6 items were excluded based on the specified inclusion/exclusion criteria. Therefore, the expert review step did not result in any unique records that were included in the final review. This has been clarified in the manuscript.

Line 153. While I agree that it doesn’t make sense to pool results from different trap types (eg EWT and tent traps) because they work through inherently different mechanisms, I think it would make sense to pool results from similar traps and attempt a meta-analysis with a random effects model to account for variability in study and trap design (eg distance between traps/HLC, trap colour, trap size, odour bait, etc). Based on the outcomes reported, it would seem feasible to calculate standardised outcome metrics (ie IRR). Since EWT had the most comparisons, this could be attempted for this trap type, and a narrative synthesis could then be used for the others. Did the authors attempt this, and what was the result?

- We investigated this possibility. Unfortunately, it is not feasible to conduct a meta-analysis, even using just EWTs. Implementing the suggested random-effects model would require controlling for individual trap design characteristics. However, the variability in trap design both within and across studies is so substantial that many combinations of EWT characteristics are represented by only a single observation. Furthermore, key information (such as the distance between traps or HLC) is missing for some cases. Together, these limitations prevent any meaningful statistical inference. We explain these limitations within the ‘Synthesis’ section.

Reviewer #2:

The manuscript offers a systematic review of all published studies comparing alternative trapping methods for molecular xenomonitoring. Objectives are clearly identified, and the methods are appropriate to achieve the goals of the study. Although the authors originally attempted to perform a meta-analysis across all published studies, this was not possible due to data scarcity and heterogeneity of experimental designs across studies.

- Thank you for reviewing the Methods.

Reviewer #3:

The objectives are clearly stated
The study design is appropriate for the objective
The selection process for articles is clearly described
The methods for synthesizing the findings support the conclusions reached
No ethical concerns

- Thank you for reviewing the Methods.

**Results**

Reviewer #1:

line 179. What is the definition of a “comparison”?

- We define a 'comparison' as an instance when the performance of an alternative trapping method was compared against the standard HLC within the same experiment. A study could have multiple experiments (e.g. testing different versions of EWTs, which were then individually compared against the standard HLC). In this case, each experiment constituted its own comparison. For experiments where multiple metrics were compared to the standard HLC (e.g. if authors compared total catch and mean hourly catch from the same experiment), this was taken to be the same comparison. We have added text to this effect in the 'Data extraction' section.

line 187. For those where the habitat was unspecified, did the papers provide GPS coordinates? This may enable inference about what habitat the collections were performed in.

- Yes, GPS coordinates were provided; however, it is not possible to reliably determine the habitat conditions at the time these studies were conducted, as such information is not consistently available. Moreover, habitats are shaped by many factors, including climate change and land-use change, that can alter conditions over time. As a result, even if we could estimate current habitat characteristics, we cannot assume they reflect the conditions present at the time of the studies.

line 194. “At least” or “over”?

- The maximum distance reported was 100 metres, so we have clarified by removing 'at least'.

line 224. For study 13, what is meant by “mean monthly biting rate” for catches that didn’t involve HLC? For those studies where “total catch” was the metric, did the authors verify that these comparisons were done 1:1? For this table, it would be useful to have more characteristics of the included studies listed. For example, which trap types the study included.

- We have added a column at the end of Table 6 indicating the trap types used in each study. In lines 151–157, we describe the study characteristics extracted from all included studies, including the ratio of alternative traps to standard HLCs. The full set of characteristics for each study and comparison is provided in the extraction dataset referenced in the “Data and Software Availability” section. It is not feasible to include all these characteristics in Table 6, as they are extensive (more than 10 variables). Additionally, some variables (e.g., distance between traps, outcome compared, statistical tests used for the comparison, number of sampling days) can vary within a single study where multiple comparisons are presented. This makes it difficult to summarise them at the study level as required for Table 6. We have included a sentence in the legend of Table 6 referring interested readers to the full extraction dataset linked in the ‘Data and software availability’ section.

Line 228. A 1:1 ratio of sampling effort or traps? If sampling effort, this would mean the traps/HLC were each performed for the same number of days and in the same quantities each day.

- This statement describes a 1:1 ratio in the number of traps rather than sampling effort, now clarified in the text. In all but two studies, sampling effort was consistent between the alternative trap and the standard HLC.

Line 245. Were there any qualities of the EWT studies that were more prevalent in the comparisons that demonstrated no sig diff/more effective? Eg did most of these include CO2, etc. May be difficult to say, but thought it might be good to tie back in Table 5. Similar question on the other studies too – did trap characteristics impact whether the trap was non inferior to HLC? For example, the Atekem study showed that black trousers were statistically better than blue or blue/black, but this isn’t mentioned. This comment also relates to an earlier comment about the possibility of implementing a random effects meta-analysis, at least on EWT studies.

- It is difficult to determine whether any specific characteristics of EWTs are associated with improved performance relative to the standard HLC. Given the substantial variation between experiments and incompleteness of data, a random-effects meta-analysis would not be appropriate (even when considering EWTs alone). Regarding the Atekem study, comparisons were limited strictly to evaluations of alternative traps against the standard HLC. We did not further compare variations of the same alternative traps with one another as this was outside the scope of the study.

Reviewer #2:

The analysis carried out is adequate considering the data available across the literature. Although the authors intended to perform a meta-analysis comparing the performance of alternative catching methods vs. HLC in different studies, this was not possible due to the scarcity and heterogeneity of data.

My only suggestion would be linking specific heterogeneity findings to individual studies (using the Ref.). This would greatly enhance the manuscript's transparency and utility. It would allow readers to understand not just that there's variation across studies, but also to identify which specific studies contribute to that variation and potentially understand why (based on their methodologies, locations, species, etc.). This level of detail would transform this review from a general summary into a more actionable reference tool for future research design and intervention planning (see below).

- As outlined in our response to Reviewer 1, who raised a similar comment linked to line 224, the full extraction dataset detailing heterogeneity across the 13 studies and 79 comparisons is available separately via Zenodo. Due to the heterogeneity being at both study- and comparison- levels, it is challenging to summarise all variables in table format by study, within the manuscript itself. We have therefore been selective, including only those variables that can be meaningfully summarised, and for those that cannot, we have provided descriptive text in the manuscript. We have formatted the entire extracted dataset to include all salient variables available in the literature included within the review, and readers have access to the full extracted dataset from all papers via the Zenodo link provided in the ‘Data and software availability’ section, which we hope will indeed be a powerful resource for those who wish to interrogate it further.

192-194. It might be useful to indicate references for the different experimental designs. For example, when the distance between the comparing trapping methods is as small as 2m, the statistical test should consider that the events are dependent; i.e., each fly is provided with a choice to land on "A" OR "B". Conversely, if the sampling methods are sufficiently far apart (e.g., 50m), catches on "A" should not affect "B". Different statistical methods should be used depending on the relative location of the sampling methods.

- Please note that we did not conduct new statistical analyses for the comparisons in this study; rather, we reported the methods and results as presented in the original papers. As noted in line 147, “statistical methods used” was one of the variables extracted during our review (see Column T of data extraction sheet available on Zenodo). In lines 232–237, we summarise the statistical tests used across studies. We agree that the choice of an appropriate statistical test depends on the experimental design, including the spatial arrangement of sampling methods. The question of at what distance blackfly collections can be considered independent is complex and would require dedicated investigation beyond the scope of this review.

194-195: Would it be useful to provide the references for the six studies in which the distance between the trapping methods was not specified? This information seems important for selecting the appropriate statistical test.

- As outlined above, we did not conduct new statistical analyses for the comparisons in this study (we have emphasised this now on line 242), however we have provided the 6 references where distance between trapping methods was not specified

200-202: As above.

- We have added references to this text. Please note that not all comparisons within a study had consistent rotation across all comparisons. Details of this can be found in the full extraction dataset.

204-207:  As above.

- We have added references to this text to specify.

227-230: References to the particular studies would be useful.

- We have added references to this text. Please note that not all comparisons within a study had consistent alternative trap Vs standard HLC ratios. Details of this can be found in the full extraction dataset

  TABLES

  Tables 3-5. Add references to the studies. See Table 6 as an example.
- We have added references to Table 3. Given this, we consider that including the same information in Table 4, which is stratified in a similar way (i.e., by country), would be redundant. For Table 5, adding a column of references (as done for Table 3) would not provide meaningful additional information, as the greatest variation occurs within studies rather than between studies. In many studies involving EWTs, multiple comparisons were made between different EWT variants as well as against the standard HLC. As a result, most studies would appear across multiple rows. A detailed descriptions of the different EWT configurations by study and comparison are available in the full extraction dataset.

Reviewer #3:

The analysis is comprehensive
Yes, the results are clearly presented
Need to work on the design or structure of the tables, the figures are good by visually judging

- We have slightly reformatted the table designs/structures but any further guidance from the editor/s on what, if anything, specifically needs changing to make publication-ready would be welcome.

**Conclusions**


-Are the conclusions supported by the data presented?

-Are the limitations of analysis clearly described?

-Do the authors discuss how these data can be helpful to advance our understanding of the topic under study?

-Is public health relevance addressed?

Reviewer #1:

The authors accurately discuss the implications of the results and do not overstate or otherwise misconstrue their findings. The authors discuss how the findings are useful in directing further research.

- Thank you for reviewing the Conclusions

Reviewer #2:

The discussion and conclusion sections provide an accurate summary of the findings. Perhaps more importantly than stating the "known", they also highlight the "unknown" and provide recommendations for future studies, such as the need for standardising methodologies.

- Thank you for reviewing the Conclusions

Reviewer #3:

Conclusions are supported by the data
The limitations are very clearly described
The discussion clearly laid out the current situation with Blackfly collection and highlights how their research is advancing the discourse
The findings and discussion are critical for advancing tools for assessing progress in onchocerciasis elimination

- Thank you for reviewing the Conclusions

**Editorial and Data Presentation Modifications?**


Use this section for editorial suggestions as well as relatively minor modifications of existing data that would enhance clarity. If the only modifications needed are minor and/or editorial, you may wish to recommend “Minor Revision” or “Accept”.

Reviewer #1:

line 69. Xenomonitoring (“foreign” monitoring) refers to the detection of pathogen DNA in haematophagous insects as a proxy for human infection. What is done in oncho programs is technically not xenomonitoring, because the goal is to estimate transmission rather than human infection. If programs had guidance around how to use whole body (rather than just head) diagnostics, then this would be xenomonitoring. Small detail, but I think important to clarify this in the manuscript.

- We agree and have changed to “surveillance”.

Line 78. Reference for the sentence that ends in biting density?

- We have clarified this to reflect it is an unknown parameter.

Reviewer #2:

INTRODUCTION

60 & 62. (Just semantics…) Although the microfilaricidal effect of ivermectin results in a reduction of transmission, I wouldn't say that it is a "preventive" drug if we consider the words "preventive" and "prophylactic" synonymous in this context. I know this is the term used in reference [1], but I'm still not convinced. Although it "prevents" transmission, it does not prevent new infections.

- We have changed to the more descriptive “microfilaricidal drugs”.

79. Similar to above, as far as I know, the prophylactic effect of ivermectin (or doxycycline, or any other drug) has not been clearly demonstrated.

- We have changed to the more descriptive “microfilaricide treatment”.

73-84. If I may, I would suggest including pruritus as an adverse effect of the HLC method. As a former collector, I can confirm that the itching afterwards is definitely unpleasant. Note that "blackfly fever" is described as a condition following high exposure to blackfly saliva. Authors can find references in the literature.

- We concur and have added text and an additional reference to highlight pruritus and additional symptoms (new reference 9).

Figure 1. Verify that index letters (a, b, c...) correctly correspond to the designated photos. I.e., there are two "c" and no "h".

- Thank you for spotting this, we have updated the figure to correct this.

RESULTS

Mexico is actually part of North America (not South America). Same for Table 3.

- We have changed South America to Americas throughout.

Reviewer #3:

The paper is well written, I recommend it for publication.

- Thank you for reviewing the Results.

**Summary and General Comments**

We thank all three reviewers for taking the time to provide such a thoughtful and comprehensive review of our work, and for their constructive comments. We have actioned the vast majority of these and provided an explanation in the few instances where it was not possible or appropriate to implement the suggested changes. Overall, these edits have helped to strengthen the manuscript.
